# In utero exposure to electronic-cigarette aerosols decreases lung fibrillar collagen content, increases Newtonian resistance and induces sex-specific molecular signatures in neonatal mice

**DOI:** 10.1007/s43188-021-00103-3

**Published:** 2021-09-06

**Authors:** Kerin M. Cahill, Manas R. Gartia, Sushant Sahu, Sarah R. Bergeron, Linda M. Heffernan, Daniel B. Paulsen, Arthur L. Penn, Alexandra Noël

**Affiliations:** 1 Department of Comparative Biomedical Sciences, School of Veterinary Medicine, Louisiana State University, Skip Bertman Dr., Baton Rouge, LA 70803 USA; 2 Department of Mechanical and Industrial Engineering, Louisiana State University, Baton Rouge, LA 70803 USA; 3 Department of Chemistry, University of Louisiana at Lafayette, Lafayette, LA 70504 USA; 4 Department of Pathobiological Sciences, School of Veterinary Medicine, Louisiana State University, Baton Rouge, LA 70803 USA

**Keywords:** Electronic-cigarettes, In utero exposures, Lung alveologenesis, Lung function, Maternal vaping

## Abstract

Approximately 7% of pregnant women in the United States use electronic-cigarette (e-cig) devices during pregnancy. There is, however, no scientific evidence to support e-cig use as being ‘safe’ during pregnancy. Little is known about the effects of fetal exposures to e-cig aerosols on lung alveologenesis. In the present study, we tested the hypothesis that in utero exposure to e-cig aerosol impairs lung alveologenesis and pulmonary function in neonates. Pregnant BALB/c mice were exposed 2 h a day for 20 consecutive days during gestation to either filtered air or cinnamon-flavored e-cig aerosol (36 mg/mL of nicotine). Lung tissue was collected in offspring during lung alveologenesis on postnatal day (PND) 5 and PND11. Lung function was measured at PND11. Exposure to e-cig aerosol in utero led to a significant decrease in body weights at birth which was sustained through PND5. At PND5, in utero e-cig exposures dysregulated genes related to *Wnt* signaling and epigenetic modifications in both females (~ 120 genes) and males (40 genes). These alterations were accompanied by reduced lung fibrillar collagen content at PND5—a time point when collagen content is close to its peak to support alveoli formation. In utero exposure to e-cig aerosol also increased the Newtonian resistance of offspring at PND11, suggesting a narrowing of the conducting airways. At PND11, in females, transcriptomic dysregulation associated with epigenetic alterations was sustained (17 genes), while *WNT* signaling dysregulation was largely resolved (10 genes). In males, at PND11, the expression of only 4 genes associated with epigenetics was dysregulated, while 16 *Wnt* related-genes were altered. These data demonstrate that in utero exposures to cinnamon-flavored e-cig aerosols alter lung structure and function and induce sex-specific molecular signatures during lung alveologenesis in neonatal mice. This may reflect epigenetic programming affecting lung disease development later in life.

## Introduction

An estimated 3.7% of adults in the United States, representing > 9 million individuals, use electronic cigarettes (e-cigs) on a regular basis [1]. E-cig usage has become increasingly popular among women of childbearing age in recent years—specifically in teenage girls [2, 3]. This is mainly due to the widespread misconception that these devices are a “healthy” alternative to traditional cigarette smoking [2, 3]. In the United States, approximately 7% of pregnant women report electronic cigarette use, despite the fact that electronic cigarette liquids (E-liquids) contain nicotine, in addition to numerous toxins, including carbonyls, volatile organic compounds, and heavy metals, which are known to impact respiratory health negatively [2, 4]. For instance, heating of the two principal components of most E-liquids, propylene glycol (PG) and glycerin, produces elevated levels of formaldehyde, a known carcinogen, which has been shown to induce wheezing, asthmatic episodes, and other respiratory symptoms [5, 6]. Moreover, the harmful effects these components may have on prenatal and postnatal development of the offspring, including effects on the lungs and overall health of the fetus and/ or neonate, are not well studied. Research pertaining to in utero e-cig exposure is relatively new, and little is known about the potentially detrimental outcomes which may result from maternal e-cig usage. Previous animal studies have found links between maternal e-cig exposure and delayed implantation; significantly decreased blood flow in the maternal uterine artery and the fetal umbilical artery; reduced body weight and length; impaired postnatal alveolar growth; and altered DNA methylation in offspring [7–10]. In addition, we have previously demonstrated that in utero exposures of mice to e-cig aerosols decreased birth weight and body length, altered lung structure, and down-regulated the expression of more than 84 lung genes related to *Wnt* signaling at birth [11]. It is, however, unknown whether in utero e-cig exposures have a persistent effect beyond birth, for instance, during lung alveologenesis.

Prenatal exposures to cigarette smoke, second-hand smoke (SHS), and/or nicotine alone have previously been shown to cause a multitude of epigenetic alterations [12–17]. Epigenetic mechanisms play a pivotal role in normal cellular development and differentiation; thus, any deviations may impede the healthy maturation of an individual [18–20]. Maternal tobacco smoking in women has been associated with DNA methylation changes [12–15]. Additionally, nicotine is a developmental toxin and has been shown in animal models to provoke multiple epigenetic changes, including altered DNA methylation in fetal lungs and histone modifications in fetal lungs and brain [16, 17]. It is therefore plausible that in utero exposure to electronic nicotine delivery systems (ENDS) devices, which can contain high concentrations of nicotine or highly concentrated nicotine salts, would also induce these detrimental changes. A recent study reported alterations to DNA methylation caused by in utero exposure of mice to e-cig aerosols, both with and without nicotine. In both cases, there was increased DNA methylation in the lungs of exposed offspring [7]. These data indicate that in utero exposures to e-cig aerosols can induce epigenetic changes in the lungs of offspring; however, the specific methylated sites or enzymes contributing to various epigenetic modifications have yet to be studied.

Further concerns when studying prenatal toxicant exposure are the effects these substances have on developing tissues and organ systems and whether these substances are impeding development. Normal prenatal and neonatal development is crucial to multiple organs, including the lungs. Lung organogenesis can be classified into four main phases: pseudo-glandular, canalicular, saccular, and alveolar [21]. The final stages of lung development involve the growth and maturation of the alveoli. In humans, alveolarization of the lungs begins at week 32 of gestation, and infants are born during the alveolar stage of development. In contrast, mice and rats are born during the saccular stage, and alveolarization occurs postnatally [22, 23]. In mice, lung organogenesis and partial lung differentiation begin at embryonic day 8 (ED8), while alveologenesis does not commence until postnatal day 5 (PND5) and continues to PNDs 21–28 [24]. During this time, alveoli develop and mature, thus drastically expanding the surface area of the lung [25]. This temporal difference in pulmonary maturation makes rodents a favorable animal model for developmental studies as translational fetal outcomes can be observed postnatally rather than preterm [24, 26, 27].

It has previously been shown that lung organogenesis is modulated by *Wnt* signaling during the development of alveoli via regulation of alveolar epithelial cell self-renewal and differentiation [25]. *Wnt* signal activation has been proven vital to the maturation of alveolar type 2 (AT2) cells, whereas inhibition of *Wnt* signaling shunts differentiation towards the AT1 sublineage during lung alveologenesis [25]. AT1 cells, which make up the vast majority of the alveolar surface, are vital to the air–blood barrier of the lungs; however, AT2 cells are also essential here, as they contribute to the synthesis and secretion of pulmonary surfactant [28]. Exposure to nicotine during these crucial stages of development, poses the threat of delaying or impairing this maturation. Interference with the process of lung alveologenesis can lead to diseases in neonates, including bronchopulmonary dysplasia [25]. In fact, exposure to nicotine-rich e-cig aerosol during the first 10 days of life causes enlarged alveoli in neonatal mice [10]. This strongly suggests that e-cig aerosols affect lung alveologenesis processes. Knowledge gaps, however, still remain related to the effects of in utero e-cig exposures on lung alveologenesis processes, lung function, and lung epigenetic modifications.

Thus, further investigations are necessary to better understand the health effects of e-cig use on vulnerable populations, e.g., pregnant women and their fetuses [29]. Based on our previous work [11], the present study was designed to test the hypothesis that in utero exposure to e-cig aerosols will lead to impaired lung alveologenesis and decreased lung function, as well as to altered epigenetic molecular signatures in the lungs of neonatal mice. Thus, at early (PND5) and medial (PND11) stages of alveologenesis in male and female Balb/c mice, we evaluated the pulmonary toxicity induced by in utero e-cig exposures; we assessed lung structure and function and surveyed the expression of genes associated with pulmonary maturation and chromatin modification.

## Materials and methods

### E-cig aerosol production

We used a 50/50 PG/glycerin cinnamon fireball-flavored E-liquid that contained 36 mg/mL of nicotine (EC Blend, Medford, OR). Cinnamaldehyde, the main flavoring chemical found in cinnamon-flavored E-liquids, has been identified in numerous non-cinnamon-labeled E-liquids on the US market [30]. The highest nicotine concentration available commercially in E-liquids in the United States is 36 mg/mL. Although American adolescents and adults preferred nicotine concentrations are 12 and 18 mg/mL, respectively [31, 32], studies based on cohorts of high school students e-cig users show that 11.7% (total participants N = 181) and 8.9% (total participants N = 478) of adolescent self-report use of e-liquid containing 18 mg/mL or greater of nicotine [33, 34]. The selection of the nicotine concentration of 36 mg/mL used in this study reflects the highest nicotine concentration available on the market and the high nicotine content used by ~ 10% of e-cig users, which replicates the nicotine intake of heavy smokers (> 1 pack of cigarette/day). This selection was also based on the nicotine content of other popular ENDS devices, including JUUL and Puff Bar, which contain nicotine salt at concentrations of 59 mg/mL.

Dams were exposed to either cinnamon-flavored e-cig aerosol or HEPA-filtered air for 20 consecutive days, covering the mouse gestational period, which last 21 days. The exposure ceased before the expected birth of offspring on the twenty-first day. Exposures for both e-cig aerosol and air control mice were conducted in 5-L whole-body exposure chambers (Scireq, Montreal, QC, Canada). To reduce the stress on the pregnant mice, we did not use individual separators, and thus, mice were free to move about the 5 L chamber during the exposure. Our e-cig aerosol exposure system was described previously [11, 35]. In brief, the e-cig aerosol was generated using a Scireq third-generation e-cig device with a battery voltage of 4.2 V and an atomizer resistance of 0.15 Ω. The system was set to generate a puff every 30 s for a duration of 3 s, which generated a 55 mL puff volume. The e-cig device was connected to a condenser and then to the whole-body chamber via a pump set at a flow rate of 2 L/min using the Scireq InExpose system (Scireq, Montreal, QC, Canada). Additional dilution air entering the exposure chamber was set at 1 L/min. The exhaust flow was set at 1 L/min. The once-daily exposures lasted 2 h. Sampling of the test atmosphere within the chamber was monitored both in real-time–with a MicroDustPro (Casella)–as well as gravimetrically, to determine total particulate matter (TPM) concentrations for each exposure session. For gravimetric measurements, test atmospheres were sampled throughout the 2-h exposure by a sampling pump (Sensidyne Gilian BDX-II air, Sensidyne, St-Petersburg, FL) set at a flow rate of 1 L/min. The aerosols were collected on 25 mm fiber filters (AP4002500, MilliporeSigma, Burlington, MA) that were weighed, before and after sampling, on a Sartorius MC5 microbalance (Sartorius, Goettingen, Germany). These exposure parameters yielded 12 air changes per hour inside the 5 L chamber, which is adequate for inhalation exposure studies. Two sets of dams were exposed in 20-day sessions. In session 1: air-exposed female mice (N = 9) gave birth to 4 litters and e-cig-exposed female mice (N = 9) gave birth to 3 litters. In session 2: air-exposed female mice (N = 7) gave birth to 2 litters and e-cig-exposed female mice (N = 11) gave birth to 4 litters. In total, 6 dams gave birth in the air group, while 7 dams gave birth in the e-cig group. The exposure parameters data for both sessions are reported in Table 1. The e-cig aerosol chemical analysis was previously performed and published elsewhere [36]. Briefly, e-cig aerosol samples were collected on site at the Inhalation Research Facility at Louisiana State University and were shipped overnight on dry ice to Enthalpy Analytical, LLC (Durham, NC). Levels of nicotine, glycerin and propylene glycol were quantified by gas chromatography with a flame ionization detector (GC-FID), and carbonyls were analyzed using EPA method TO-11A based on high performance liquid chromatography (HPLC). All these analyses were made by Enthalpy Analytical, LLC and results are shown in Table 2.

**Table 1:** Air and e-cig aerosol exposure characterization data

Parameters	Session 1		Session 2	
	AIR	E-CIG	AIR	E-CIG
Aerosol concentration (mg/puff)	–	0.165 ± 0.11	–	0.164 ± 0.09
Temperature (°C)	26.3 ± 2.5	25.1 ± 1.9	26.4 ± 3.1	26.0 ± 2.1
Humidity (%RH)	77.7 ± 11.0	73.0 ± 8.5	78.7 ± 7.9	82.2 ± 7.6

Data are expressed as mean ± standard deviation (SD)

**Table 2:** Chemical profile of a cinnamon-flavored e-cig aerosol generated using a third-generation e-cig device operating with a voltage set a 4.2 V and atomizer resistance of 0.15 Ω

Compounds	Mean concentration (µg/puff)
Nicotine	567
Glycerin	11,800
Propylene glycol	4900
Acetaldehyde	0.386
Acetone	0.354
Benzaldehyde	0.630
Crotonaldehyde	0.104
Formaldehyde	0.021
m&p-tolualdehyde	1.460
Propionaldehyde	0.049

### Animal exposure protocols

We exposed adult BALB/c female mice (Charles River, Wilmington, MA), aged between 11 and 13 weeks, to either e-cig aerosol or HEPA-filtered air for 2 h a day for 20 consecutive days. Mating took place at a 2:1 female-to-male ratio during the first 5 days of exposure. Dams of both air and e-cig groups gave birth within 4 days of each other in both sessions. This resulted in dams being exposed for 16 to 20 days of their pregnancy, which covers the lung organogenesis and lung differentiation periods which begin at embryonic day 8 (ED8) [24]. At birth (PND0), the number of live and/or stillborn neonates was documented, as well as the birth weight of the offspring. Individual neonates were weighed and measured crown-to-rump at PND5. Dams were kept with their litters allowing for proper maternal care and lactation until the neonates were necropsied. Dams were necropsied following the neonate necropsies. At PND5 and PND11, offspring were euthanized with Beuthanasia-D (Schering-Plough, NJ) via an intraperitoneal injection followed by decapitation. Blood was collected and pooled by litter, and lungs were excised and either put on dry ice, stored in RNAlater or formalin-fixed, for subsequent analysis. Dams were sacrificed on PND12 via an intraperitoneal injection of Beuthanasia-D. All mice were housed in an AAALAC-approved animal care facility at the School of Veterinary Medicine of the Louisiana State University under a 12-h light/dark cycle (from 6:00 am to 6:00 pm). The mice had access to water and food ad libitum, except during the 2-h exposure periods. Mice were housed and handled in accord with the NIH Guide for the Care and Use of Laboratory Animals. All procedures and protocols were approved by the Louisiana State University Institutional Animal Care and Use Committee.

### Preparation of lungs slides and histopathological evaluation

Lungs of the PND5 and PND11 offspring were inflated and pressure-fixed with intratracheally-instilled 10% formalin. Lungs were sectioned, cut, and stained by standard hematoxylin and eosin (H&E) staining procedures. Lung histopathological evaluation was performed by a board-certified veterinary pathologist. Scoring of neonatal lung tissue by pathologists are routinely done in biomedical research [37–39]. Lung tissue was scored for aeration, fetalization, and inflammation. Aeration was defined as the percentage of expansion of the lungs (1 = 0–40%; 2 = 40–60%; 3 = 60–80%; and 4 = 80–100%). Fetalization was defined as the degree of fetal appearance of the lungs (0 ~ to an adult lung → 3 ~ to a fetal lung). Inflammation was defined based on the presence of peri-bronchial inflammatory cells (0 = absent, 1 = minimal, 2 = mild, and 3 = moderate).

### Lung fibrillar collagen content analysis by multiphoton imaging

We used second harmonic generation (SHG) microscopy images to determine the fibrillar collagen content of the lungs. The instrument used was a Leica SP5 resonant scanning multiphoton confocal inverted microscope (Leica Microsystem), coupled to a Spectra Physics Mai-Tai tunable pulsed near-IR laser (690–1040 nm), which enabled SHG and two-photon excited fluorescence (TPF) imaging. We used the following conditions for all SHG imaging data collection: excitation wavelength of 860 nm with a pulse width of 70 fs produced at 84 MHz repetition rate. SHG images were acquired in a noncontact imaging mode where the laser pulses were focused onto the lung tissue specimens through a 20X, 0.70 NA air objective (Obj. HC PL APO 20x/0.70 CS, Leica), attenuated to approximately 50 mW at the objective focus in the SHG imaging experiments. The SHG signal and TPF were collected in the backward direction using the same objective by selecting a filter cube (680 nm Short Pass filter) equipped with 320–430 nm range band-pass filter (> 90% transmission) for SHG and 486–506 nm bandpass filter for TPF, detected by highly sensitive photomultiplier tube (PMT) detectors. For laser scanning control and image acquisition we used the LAS X software (Leica Microsystems). Image were captured at a 400 Hz scan speed per line (size: 1024 X 1024 pixels). SHG and two-photon excited fluorescence are represented in pseudo color, green and red, respectively. All images were analyzed employing ImageJ (NIH, Bethesda, MD) software for quantitative image analysis by calculating the fraction of pixels with SHG signal (green pixels) to total sum of pixels containing both green and red pixels.

### Pulmonary function testing of offspring (PND11)—Forced Oscillation Technique

Pulmonary function of offspring (*n* = 4–6 per group) was measured invasively at PND11 with the *flexiVent* system using the FX Module 1, which is the module for neonate mice, which allows to perform lung function testing in neonate mice weighing > 3 g (Scireq, Montreal, Canada). This time point (PND11) was chosen as this was the first-day offspring met the cannula intubation requirements for the device. Mice were anesthetized by subcutaneous injection of a ketamine and xylazine cocktail. Once unconscious, the animals were tracheostomized, and a blunt needle (21 G, 0.5″, part # B21-50 SAI Infusion Technologies) was placed in the airway. Animals were then connected to the flexiVent system to collect lung function data based on a ‘mouse mechanics scripts’. This script conducts a series of manoeuvres in sequential loops, including, in this order: 1- Deep inflation; 2- Snap-shot-150 [collecting data for total resistance (R) and compliance (C)]; and 3- quick prime-3 [collecting data for Newtonian resistance (RN)]. Only measurements with a coefficient of determination of > 0.95 were accepted. Parameters of respiratory system resistance, compliance, and Newtonian resistance were measured and calculated using the single compartment and constant phase models. At least 5 measurements for each of the lung function parameters were recorded and averaged for each mouse. After all the measurements were completed, animals were euthanized using Beuthanasia-D (Schering-Plough, NJ) via intraperitoneal injection.

### Genotyping PND5 and PND11 offspring for sex

Prior to lung RNA extraction, sections taken from the same lung tissue samples were sent to Transnetyx Inc. (Cordova, TN) for automated genotyping with the help of a sample collection kit provided by the company. The genotyping results showed that the female-to-male ratio was ~ 0.6 in both air and e-cig groups.

### Lung RNA extraction

Lungs were collected from offspring (PND5 and PND11) and were placed in RNAlater to preserve the tissue for RNA extraction. RNA was extracted from samples using RNAeasy Mini Kit (QIAGEN, USA) as per the manufacturer’s instructions. Following the extractions, we used a NanoDrop ND-1000 Spectrophotometer (NanoDrop, Wilmington, DE) to verify the purity and quantity of lung RNA obtained.

### Preparation of cDNA

After treatment of extracted RNA with DNase, the RT2 First Strand Kit (Qiagen 330401) was used in order to reverse-transcribe total RNA. The resulting cDNA was diluted with high-quality RNase-free water to a volume of 111 μl. Finally, 102 μl of the cDNA sample was combined with RT2 SYBR Green qPCR Master mix (Qiagen 330,503).

### Gene expression analysis—RT2-PCR arrays

We used RT2 profiler PCR arrays (Qiagen, USA), according to the manufacturer’s instructions, to assess the expression of 84 *Wnt* Signaling genes (Catalog number 330231 PAMM-043ZA) and 84 Epigenetic Chromatin Modification genes (Catalog number 330231 PAMM-085ZR) in the lungs of offspring collected at PND5 and PND11. We calculated gene expression and fold changes via the ΔΔCt method with the Qiagen GeneGlobe data analysis software.

### Gene expression analysis—qRT-PCR

We performed quantitative PCR on cDNA samples of lung homogenates from the dams and their respective offspring at PND5 to assess the expression levels of selected inflammatory genes, as described [11]. The cDNA was prepared from 2 µg of RNA with a High-Capacity cDNA kit (Applied Biosystems, P/N 4374966). The qPCR reaction mixture contained 5 µL cDNA (diluted 1:10 with nuclease-free water), 12.5 µL iTaq Universal Probe Supermix (BioRad), 6.5 µL H_2_O, and 1 µL FAM-labeled TaqMan™ probe (Applied Biosystems). We carried out amplification with a 7300 Real-Time PCR System (Applied Biosystems) using the standard TaqMan protocol: 50 °C (2 min); 95 °C (10 min); and 40 cycles of 95 °C (15 s), 60 °C (60 s). Relative gene expression (ΔΔC_T_) for each gene was normalized to hypoxanthine guanine phosphoribosyltransferase (Hprt1) expression and reported as fold change over control [(2^−ΔΔCT^)].

### Gene network and canonical pathway analysis

Gene expression data gathered from the *Wnt* Signaling Pathway and Epigenetic Chromatin Modification RT2 Profiler Arrays were analyzed using QIAGEN Ingenuity Pathway Analysis (IPA), Reactome Knowledgebase Pathway Analysis, and DAVID Bioinformatics Database [40, 41]. This allowed us to determine the most significantly enriched canonical pathways and networks of gene interactions.

### Statistical analyses

Results were analyzed using either: (1) the Student t-test for pairwise comparisons or (2) ANOVA followed by the Tukey’s test for multiple comparisons. Outcomes are presented as mean ± standard error of the mean (SEM). Statistical analyses were performed using GraphPad Prism 8 Software (GraphPad Software, San Diego, CA). Results with a *p* < 0.05 were considered significant.

## Results

### In utero e-cig exposure decreases offspring birth weight through PND5

**Fig. 1:**
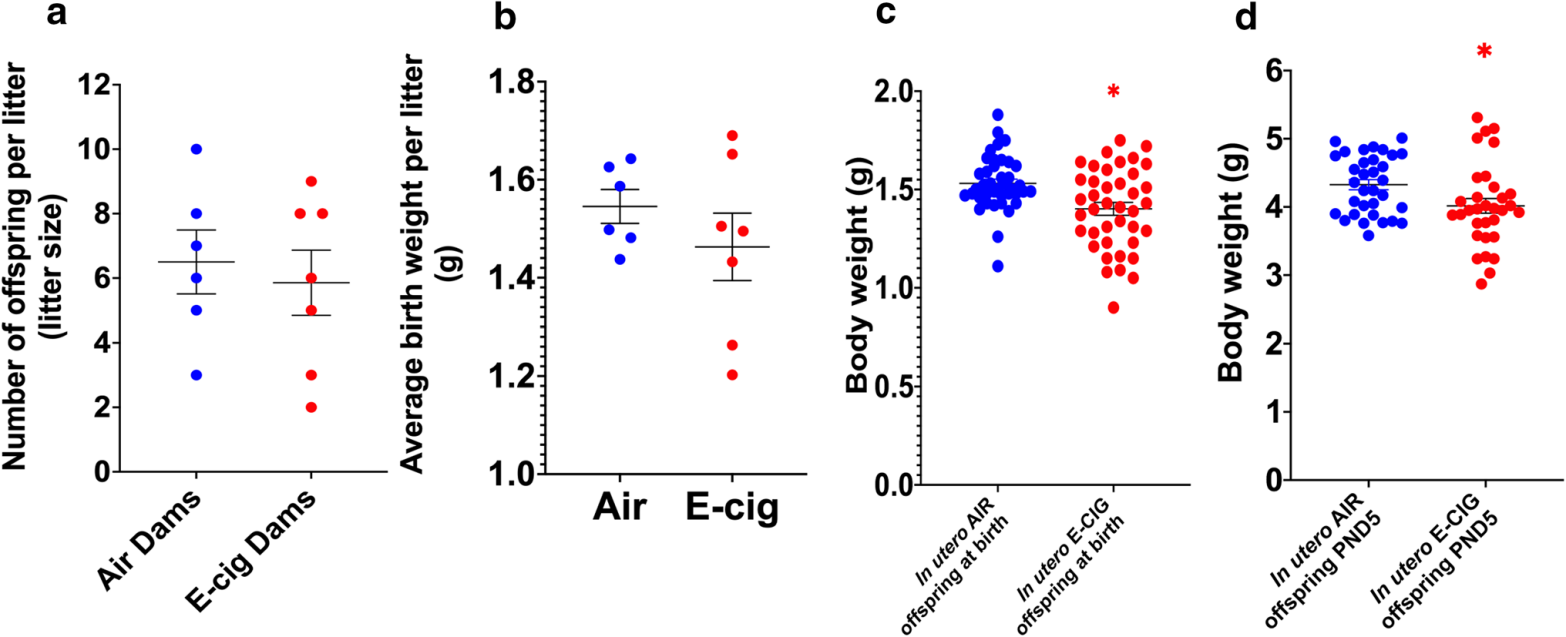
In utero e-cig exposure decreases offspring birth weight through PND5. Litter size of dams exposed either to filtered-air or e-cig aerosol **a**. Average litter birth weights per dam. **b** Body weight of mice exposed in utero either to filtered-air or e-cig aerosol recorded at PND0 **c** and PND5 **d**. PND0 N = 40 per group. PND5 N = 32–33 per group. Data are expressed as mean ± SEM. Student t-test, *p < 0.05: statistically different from in utero air group

Pregnancy outcomes were measured and recorded at birth to determine whether prenatal exposure to e-cig aerosol led to unfavorable pregnancy outcomes such as still births or low birth weights. Although there were no statistically significant differences in stillbirths (0 in the air group vs. 1 in the e-cig group), birth weights of newborns subjected to e-cig aerosol in utero were significantly decreased (*p* < 0.05) compared with weights of their air-exposed counterparts—despite litter sizes and average litter birth weights being comparable (Fig. 1a, b, c). This reduced body weight was sustained through PND5 (*p* < 0.05) (Fig. 1d). At PND5, the length of the offspring, measured crown-to-rump, was similar between the air (3.91 ± 0.26 cm) and e-cig (3.86 ± 0.25 cm) exposed groups.

### In utero e-cig exposure decreases lung fibrillar collagen content in PND5 offspring

**Fig. 2:**
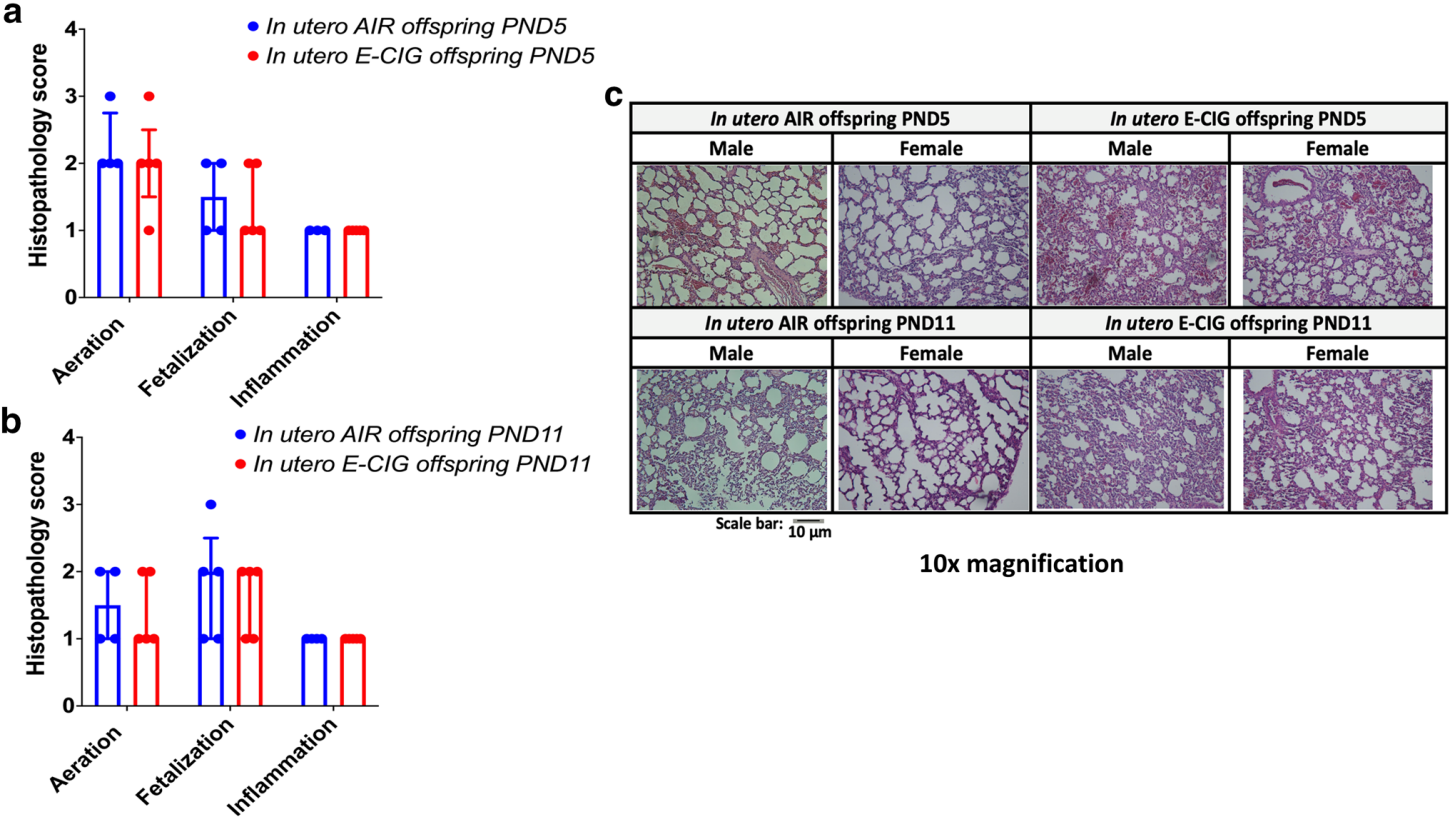
Histopathological assessment of neonatal mice lung tissue revealed no difference between groups. Mice exposed in utero to e-cig aerosol did not exhibit significant differences in aeration percentage, fetalization, or inflammation scores when compared with the in utero air group at PND5 **a** and PND11 **b**, **c** Representative microscopy images of H&E stained lungs (right caudal lobe) from each group (10 × magnification; scale bar = 10 µm). Aeration was defined as the percentage of expansion of the lungs (1 = 0–40%; 2 = 40–60%; 3 = 60–80%; and 4 = 80–100%). Fetalization was defined as the degree of fetal appearance of the lungs (0 ~ to an adult lung → 3 ~ to a fetal lung). Inflammation was defined based on the presence of peri-bronchial inflammatory cells (0 = absent, 1 = minimal, 2 = mild, 3 = moderate and 4 = marked). N = 4–5 per group; data are expressed as median ± interquartile range

**Fig. 3:**
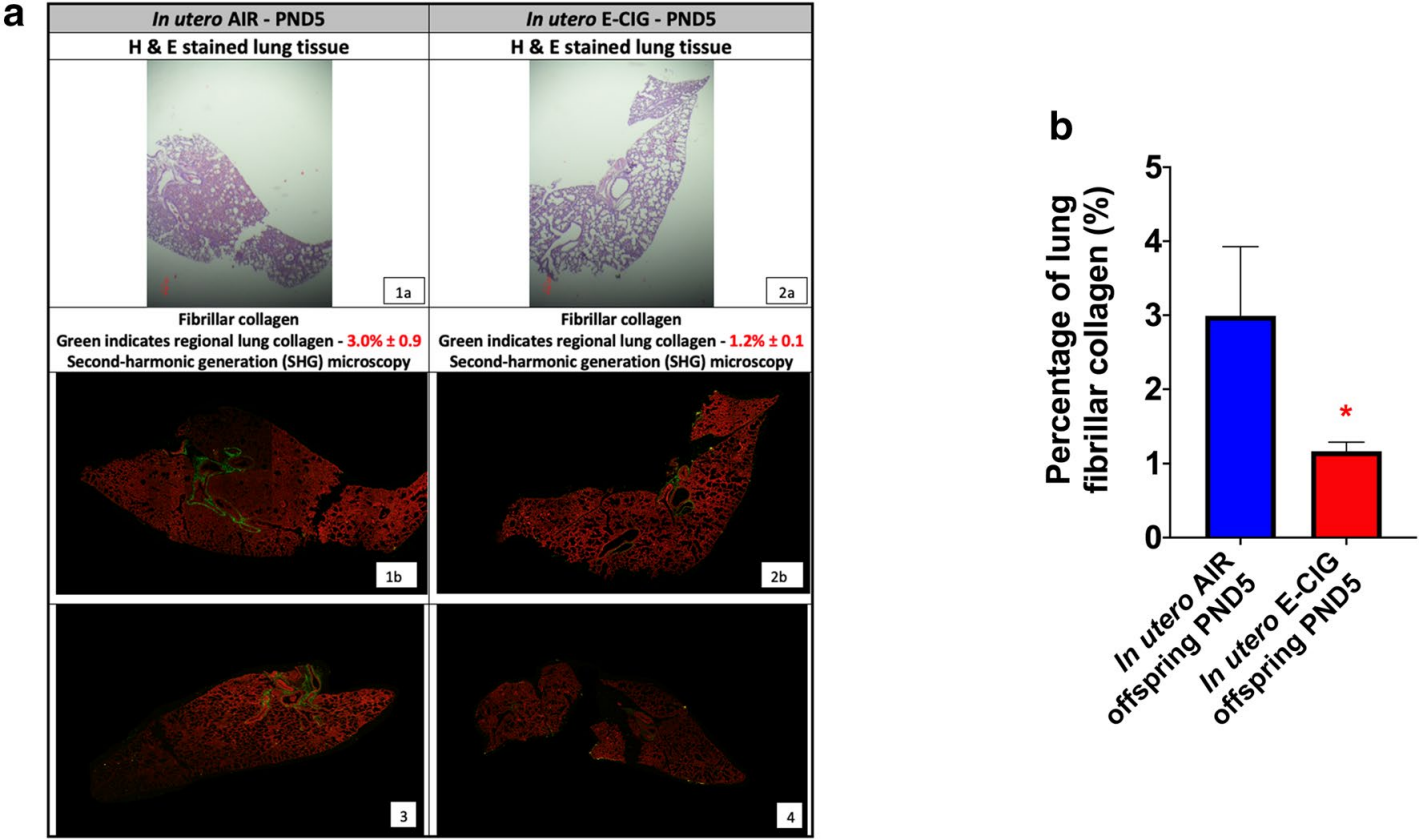
In utero e-cig exposure decreases lung fibrillar collagen content in PND5 offspring Fibrillar collagen content of mice exposed in utero either to filtered-air or e-cig aerosol examined at PND5. **a** Representative microscopy images of H&E stained lungs (right caudal lobe) (1a: air female offspring; 2a: e-cig male offspring) along with images of the same lung section (1a and 2a) obtained by SHG microcopy for each group (1b: air female offspring; 3: air male offspring; 2b: e-cig male offspring; 4: e-cig female offspring). SHG (fibrillar collagen) and two-photon excited fluorescence are represented in pseudo color, green and red, respectively. **b** Quantification of the lung fibrillar collagen content. N = 4 per group; data are expressed as mean ± SEM. Student t-test, *p < 0.05: statistically different from in utero air group

To determine whether in utero e-cig exposure affected lung structure, we conducted a histopathology assessment of lung slides at both PND5 and 11 (Fig. 2) and investigated the fibrillar collagen content in those lung slides at PND5, via SHG microscopy (Fig. 3). This time-point was selected for collagen content analysis since the maximum levels of interstitial collagen are found at PND7, a critical window in alveologenesis corresponding to secondary septation [25, 27]. No significant differences were observed in the histopathology scores for the aeration, fetalization, and inflammation criteria (Fig. 2). These results were confirmed by morphometric analyses of the fraction of septal tissue in the parenchyma, where no significant difference was observed between the air (PND5: 40.0% ± 0.04) (PND11: 43.9% ± 0.01) and the e-cig groups (PND5: 41.4% ± 0.05) (PND11: 43.0% ± 0.08). Notwithstanding, we found that offspring exposed to e-cig aerosol in utero exhibited a significant decrease (*p* < 0.05) of total lung fibrillar collagen content when compared to the lung tissue of neonates exposed to filtered air (Fig. 3a, b). Lung fibrillary collagen forms the network of fibers that plays a vital role in alveolar formation [42].

### In utero e-cig exposure modulates the maternal–fetal interface in terms of inflammatory lung makers at PND5

**Fig. 4:**
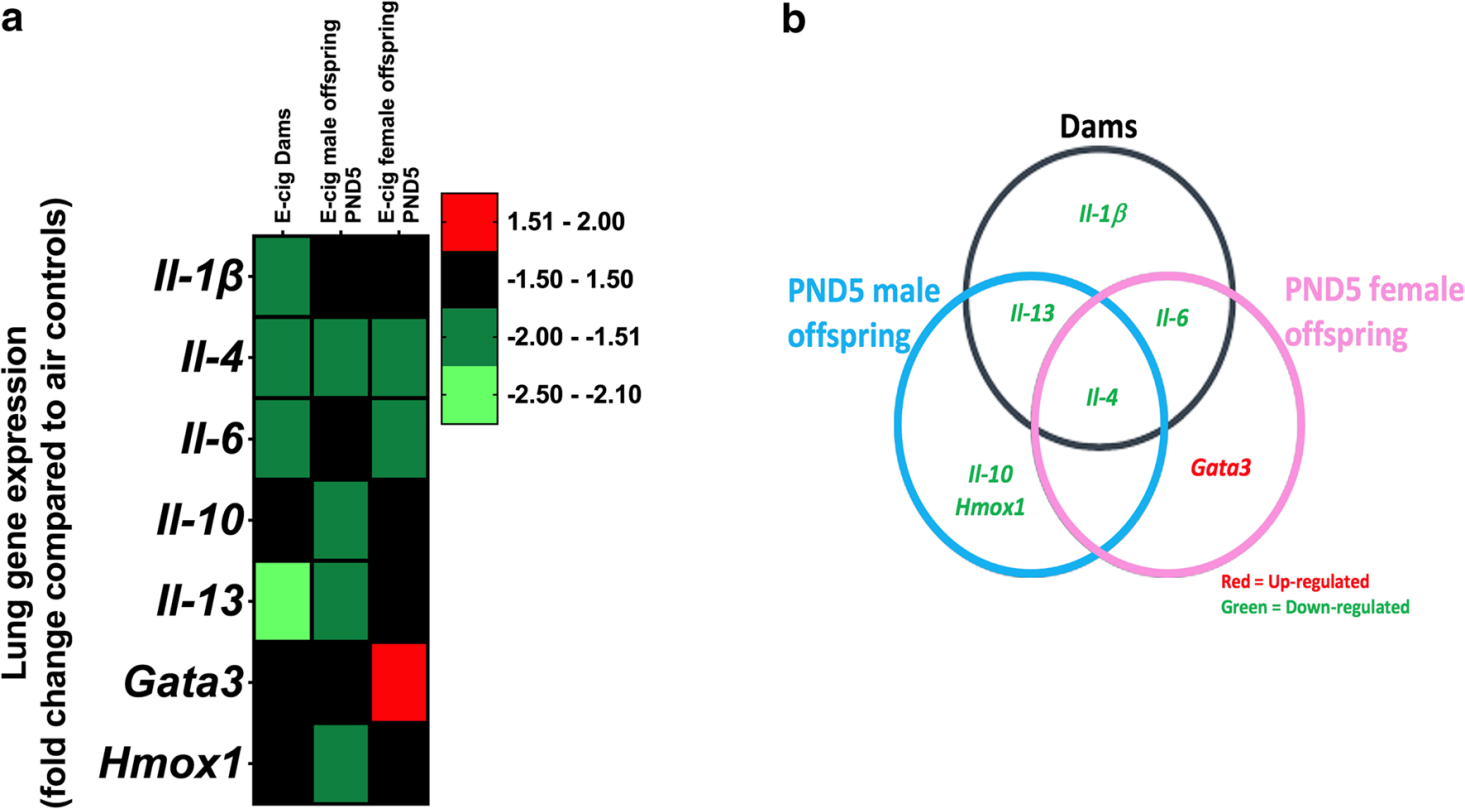
In utero e-cig exposure modulates the maternal–fetal interface in terms of lung inflammatory makers at PND5. **a** Heatmap displays qPCR results expressed in fold-change compared to respective air control group. **b** Venn diagram showing common dysregulated genes in the various groups. N = 4 mice per group for the PND5 offspring; N = 5–6 per group for the dams. Data are expressed as mean. Data >  ± 1.5-fold-change were considered significant.

To elucidate how in utero e-cig exposures affect pulmonary inflammation markers in both dams and offspring, we determined the expression of selected inflammation-related genes at PND5. Four genes were significantly down-regulated in the dams (*Il-1ß*, *Il-4*, *Il-6*, *Il-13*). Four genes (*Il-4*, *Il-10*, *Il-13*, *Hmox1*) were significantly down-regulated in the PND5 male offspring. Two genes (*Il-4*, *Il-6*) were significantly down-regulated, and one (*Gata3*) was significantly upregulated in the PND5 female offspring (Fig. 4). Only the downregulation of *Il-4* was common to dams and PND5 male and female offspring (Fig. 4b).

### In utero e-cig exposure affects lung function in PND11 offspring

**Fig. 5:**
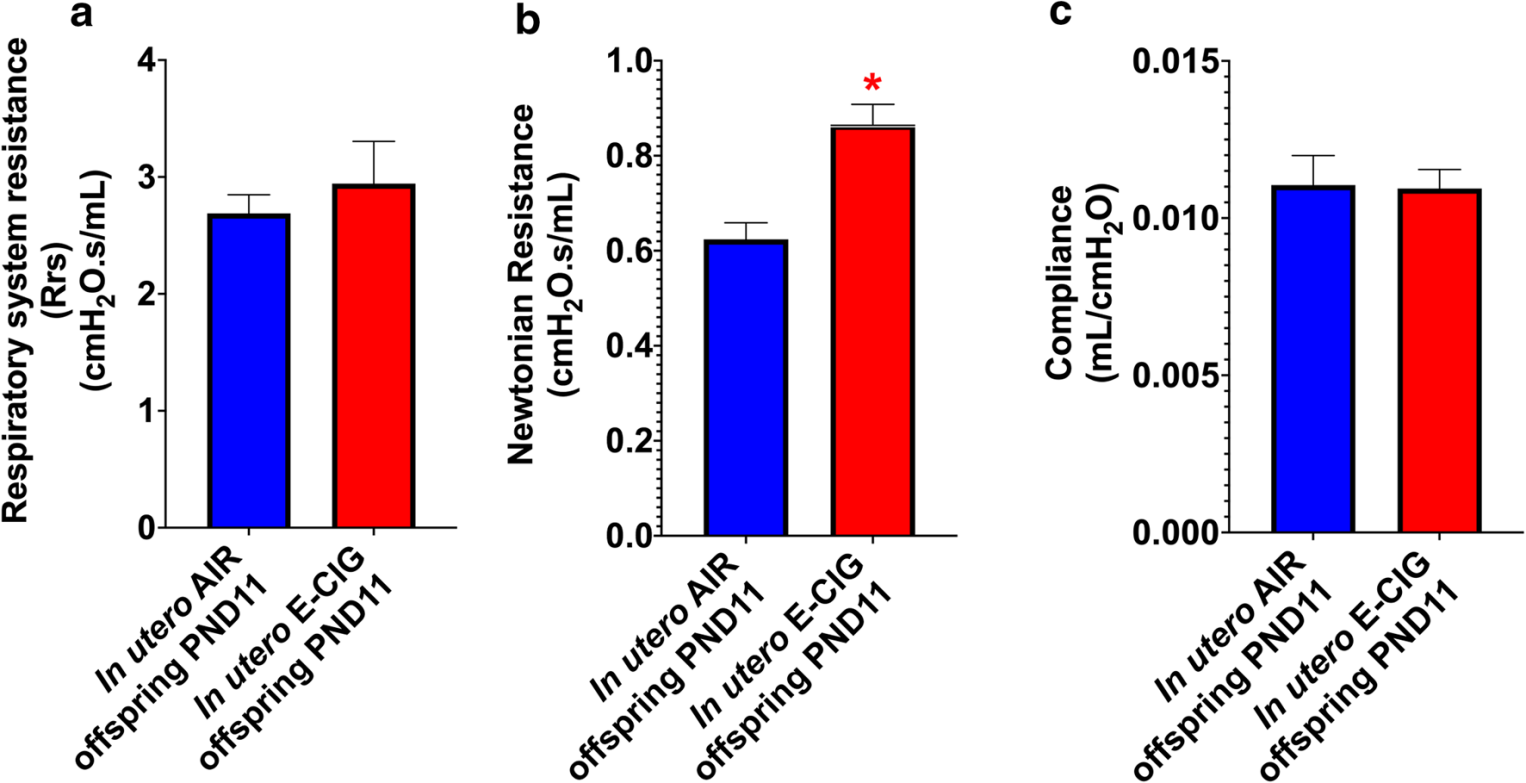
In utero e-cig exposure increases Newtonian lung resistance in PND11 offspring. **a**, **b** Respiratory system resistance and Newtonian lung resistance of mice exposed in utero either to filtered-air or e-cig aerosol recorded at PND11. **c** Lung compliance of mice exposed in utero either to filtered-air or e-cig aerosol recorded at PND11. N = 4–6 offspring per group. The offspring came from 3 different litters for the air group and from 4 different litters for the e-cig group. Data are expressed as mean ± SEM. Student t-test, *p < 0.05: statistically different from in utero air group

To determine whether in utero e-cig exposures affect neonatal lung function, we performed invasive pulmonary function testing at PND11. Based on the *flexiVent* data, we found no significant differences in the values for respiratory system resistance and compliance between the mice exposed in utero to e-cig aerosol or to HEPA-filtered air (Fig. 5a, c). Exposure to e-cig aerosol, however, significantly increased (*p* < 0.05) the Newtonian resistance of the PND11 offspring (Fig. 5b). Newtonian resistance represents the resistance of the conducting airways; therefore, a noticeable increase of this parameter indicates a narrowing of the airways.

### In utero e-cig exposure imprints sex-specific molecular signatures in lung tissue

**Fig. 6:**
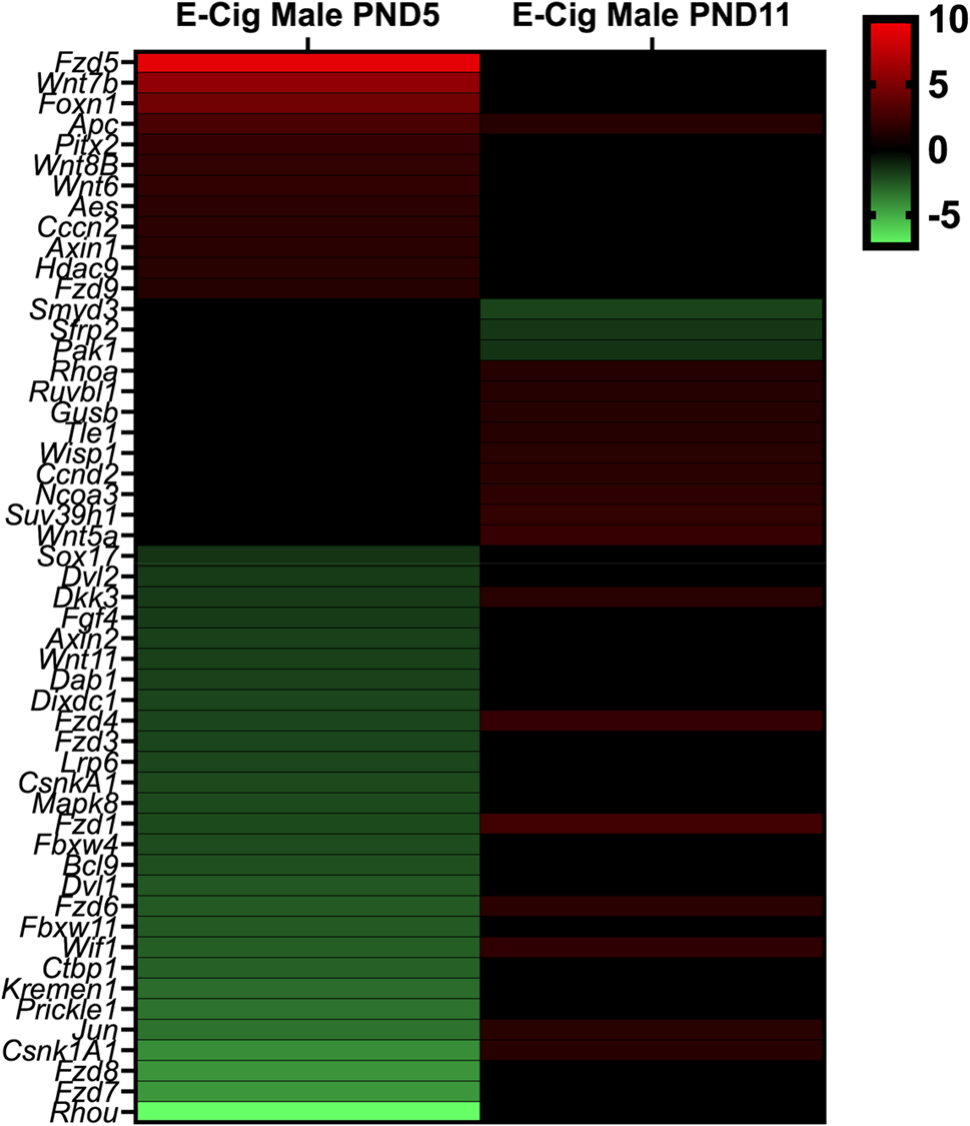
Dysregulation of *Wnt* pathway and epigenetic chromatin modification genes in male mice exposed in utero to e-cig aerosols. Male mice exposed in utero to e-cig aerosol exhibited dysregulation of a number of genes associated with the *Wnt* signaling pathway and epigenetic chromatin modification at both PND5 and 11. N = 3–4 per group. Data are expressed as mean fold-change compared to respective in utero air control group. Data >  ± 1.5-fold-change were considered significant

**Fig. 7:**
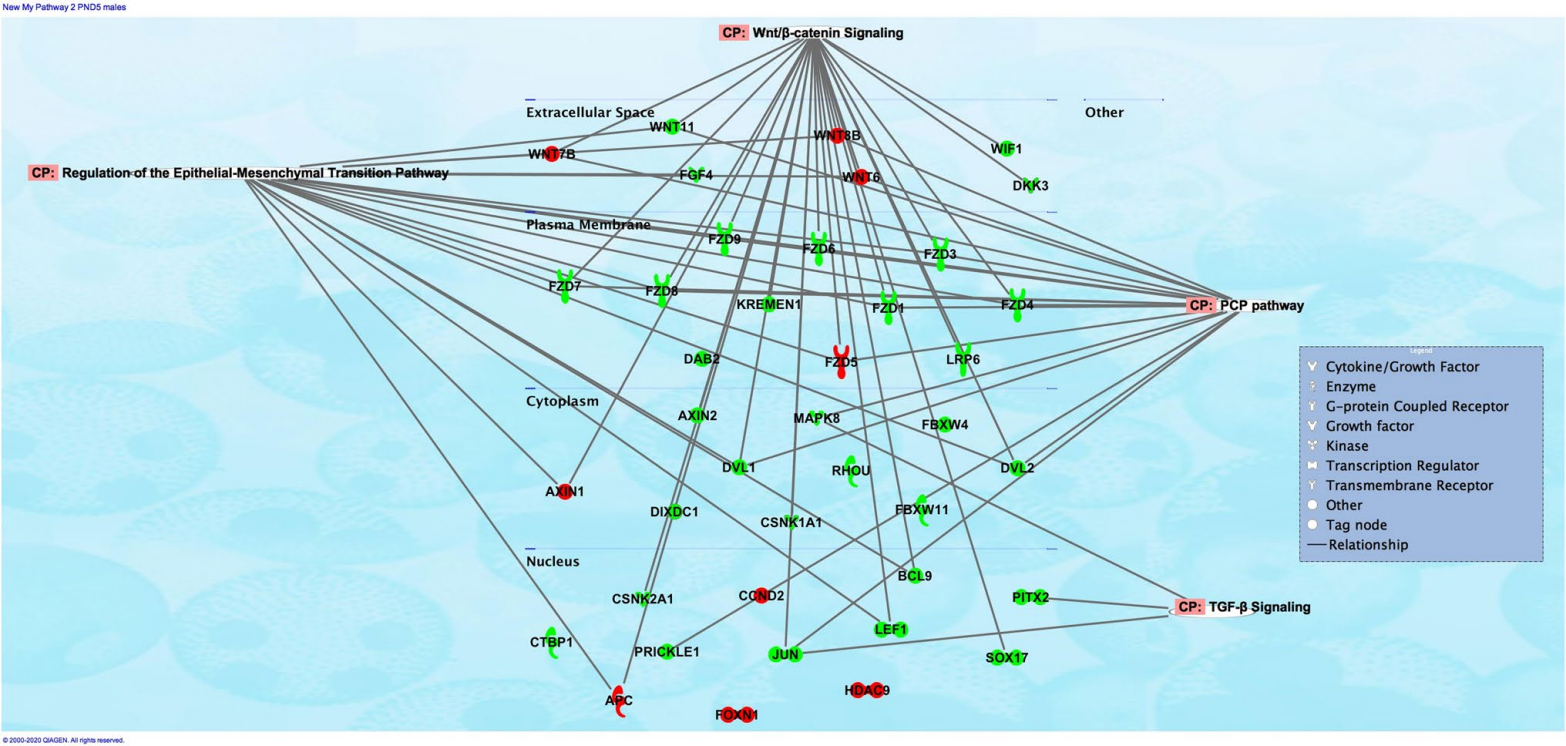
Pathway analysis revealed that networks of genes dysregulated by the in utero e-cig exposure were associated with regulation of epithelial-mesenchymal transition pathway in PND5 male offspring. At PND5, male offspring exposed in utero to e-cig aerosol exhibited dysregulation of genes associated with *Wnt* signaling, regulation of epithelial-mesenchymal transition (EMT) pathway, PCP pathway, and TGF-B signaling, when compared to the respective in utero air control group. Genes in red show up-regulation, while genes in green show down-regulation

To reveal the possible molecular mechanisms associated with altered lung development following in utero e-cig exposures, we analyzed the expression of 84 *Wnt* signaling genes, as active *Wnt* signaling during lung alveologenesis and maturation has previously been demonstrated [25]. Additionally, due to previous evidence that in utero e-cig exposures increase DNA methylation in the lungs of offspring [7], we analyzed the gene expression of 84 epigenetic chromatin modification genes in the lungs of the neonates. At PND5, in utero e-cig aerosol exposures dysregulated one epigenetic chromatin modification gene and 39 *Wnt* signaling-associated genes in males. In contrast, at PND11, a total of 20 genes were dysregulated, including 16 from the *Wnt* pathway and 4 related to epigenetics (Fig. 6). There were 7 common dysregulated genes at both PND5 and 11 in male offspring (Fig. 6). In female offspring, at PND5, 61 genes of the *Wnt* pathway and 60 genes associated with epigenetic chromatin modifications were dysregulated by the in utero e-cig aerosol exposures (Fig. 7). In female offspring, there were 24 genes commonly dysregulated at PND5 and 11 (Fig. 7). At PND11, female offspring exposed in utero to e-cig aerosols had 10 *Wnt* pathway genes and 17 epigenetic chromatin modification genes that were dysregulated (Fig. 7).

**Fig. 8:**
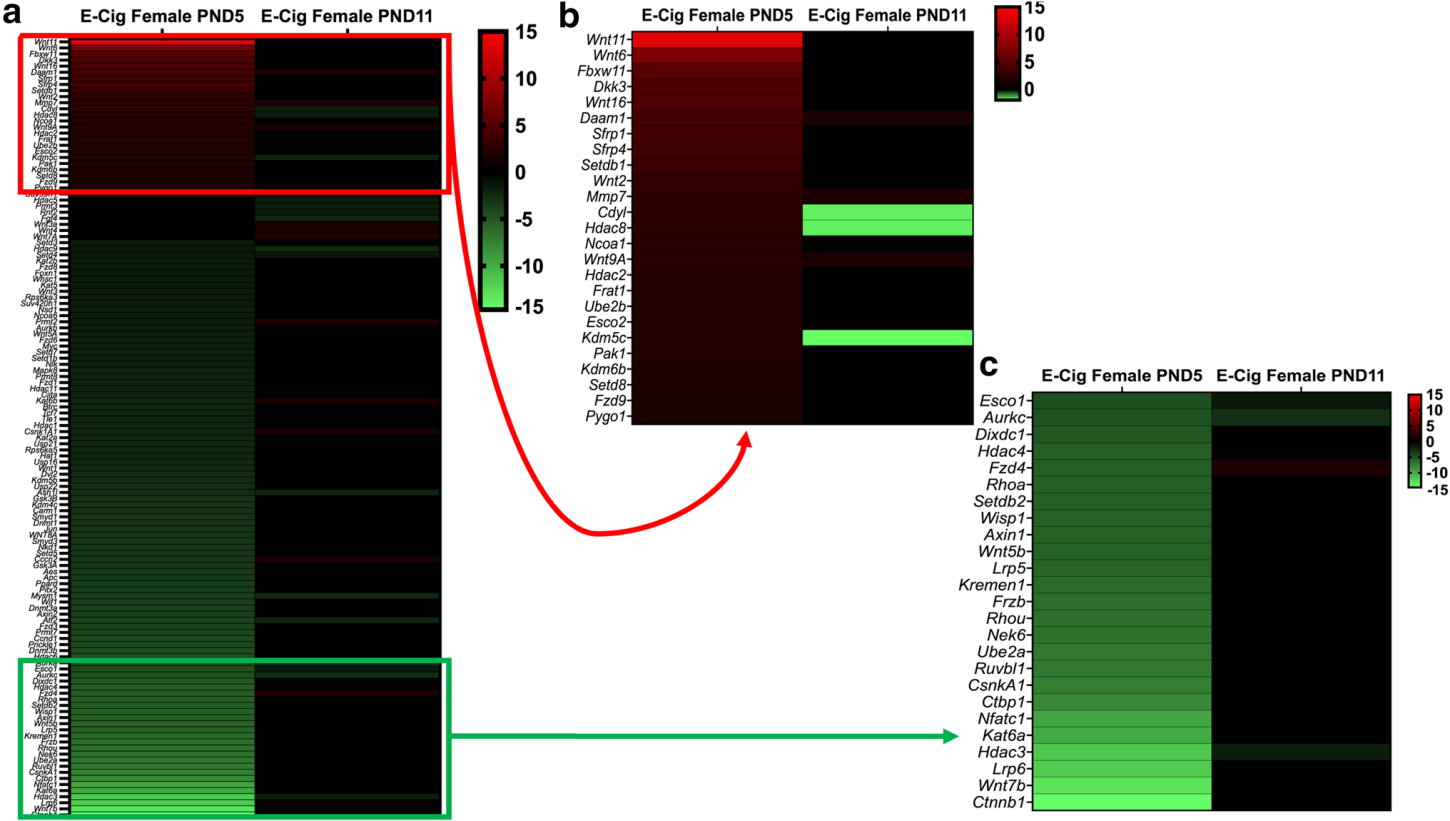
Dysregulation of *Wnt* pathway and epigenetic chromatin modification genes in female mice exposed in utero to e-cig aerosols. **a** Female mice exposed in utero to e-cig aerosol exhibited dysregulation of a number of genes associated with the *Wnt* signaling pathway and epigenetic chromatin modification at both PND5 and 11. **b** Zoom-in on the top 25 most up-regulated genes. **c** Zoom-in on the 25 most down-regulated genes. N = 4 per group. Data are expressed as mean fold-change compared to respective in utero air control group. Data >  ± 1.5-fold-change were considered significant

**Fig. 9:**
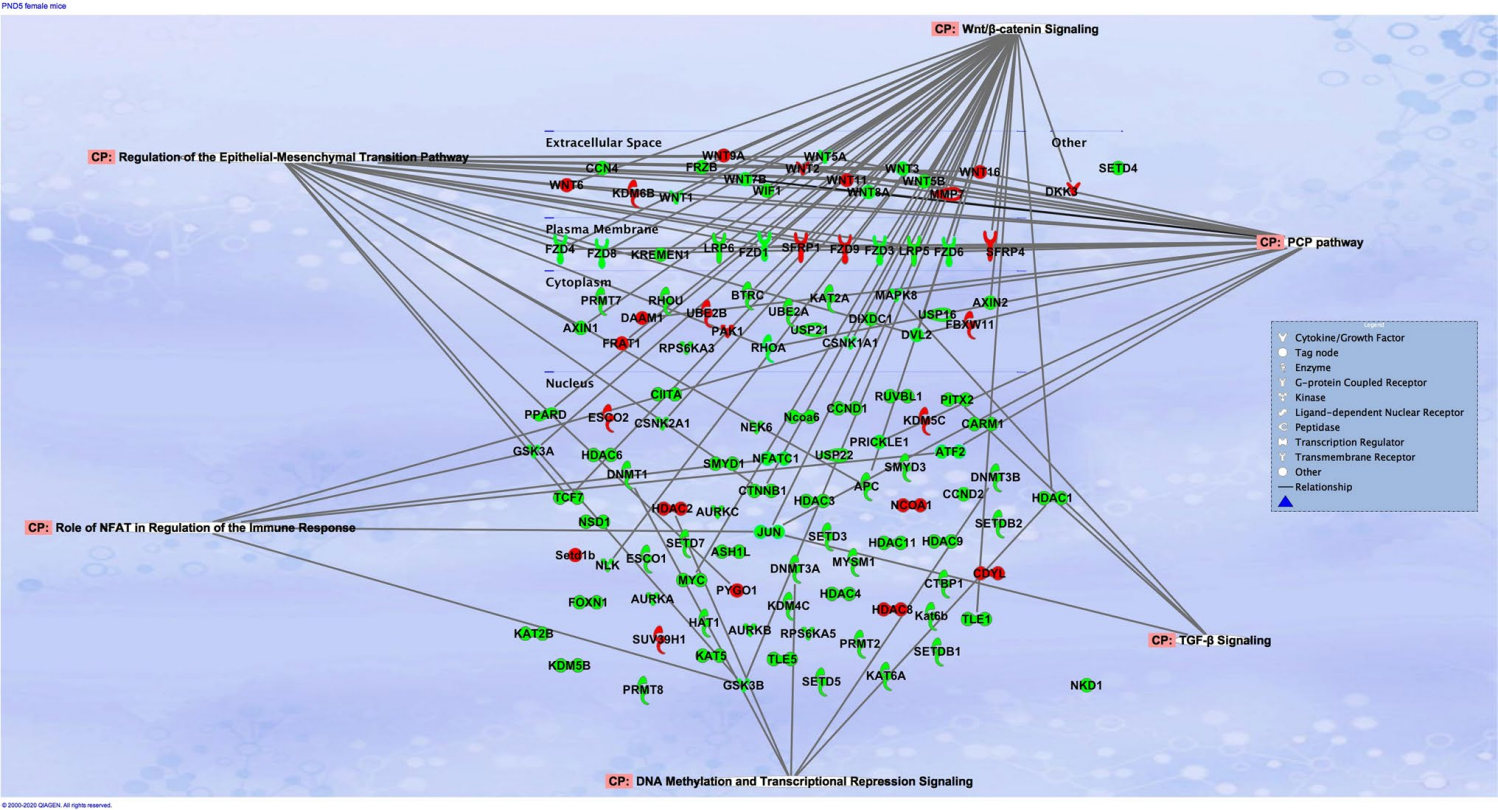
Pathway analysis revealed that networks of genes dysregulated by the in utero e-cig exposure were associated with the role of NFAT in regulation of immune responses in PND5 female offspring. At PND5, female offspring exposed in utero to e-cig aerosol exhibited dysregulation of genes associated with *Wnt* signaling, regulation of epithelial-mesenchymal transition (EMT) pathway, PCP pathway, the role of NFAT in regulation of the immune response, and TGF-B signaling, when compared to the respective in utero air control group. Genes in red show up-regulation, while genes in green show down-regulation

**Fig. 10:**
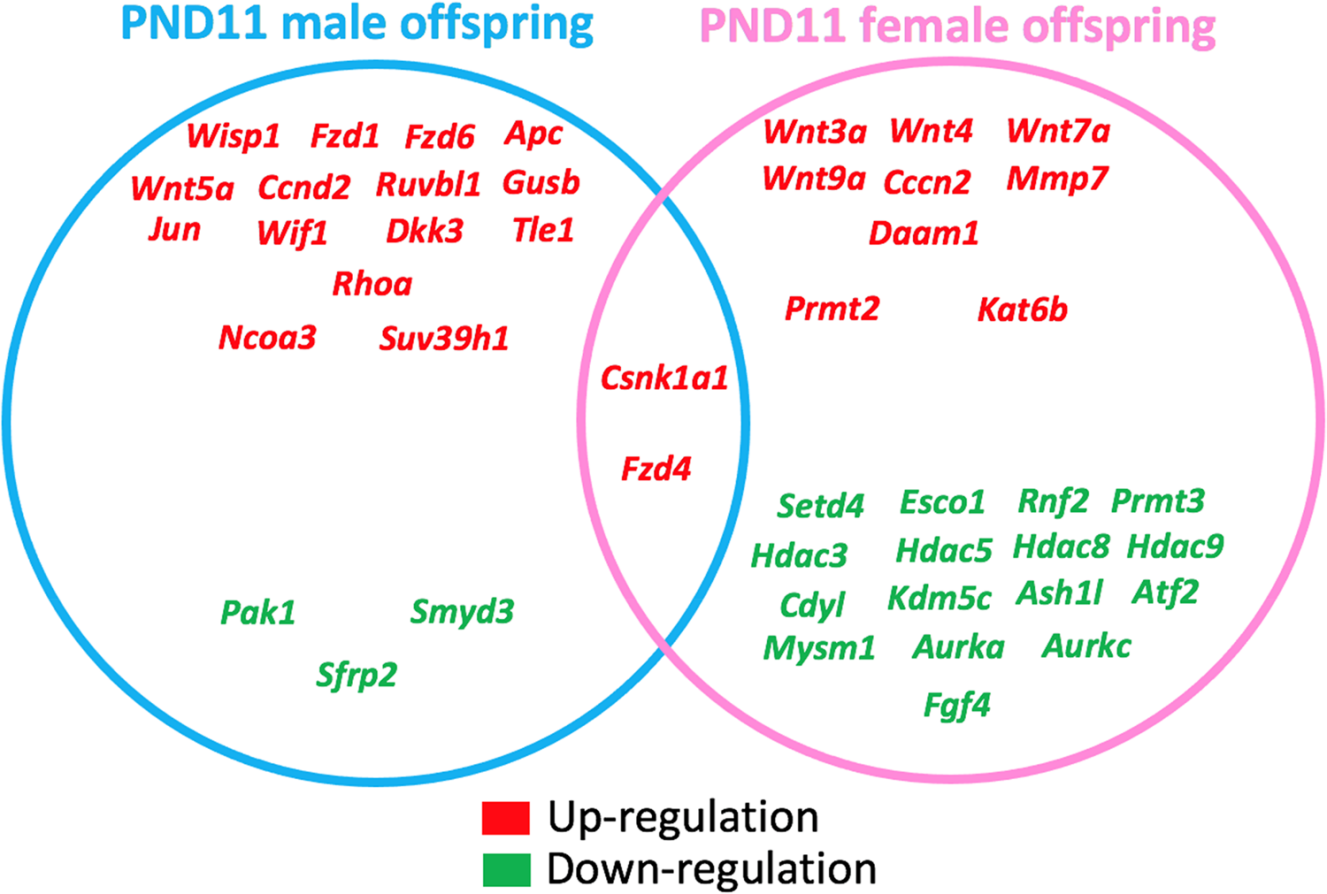
Sex-specific dysregulation of *Wnt* pathway and epigenetic chromatin modification genes in mice exposed in utero to e-cig aerosols at PND11. In male offspring, a total of 20 genes were dysregulated at this time point and 2 genes were in common with the female offspring. The 18 male-only dysregulated genes included the up-regulation of 13 genes from *Wnt* signaling and 2 genes associated with epigenetics (*Ncoa3* and *Suv39h1*), in addition to the down-regulation of 1 gene related to *Wnt* (*Sfrp2*) and 2 genes linked to epigenetic modifications. In the female offspring, the 25 female-only dysregulated genes included the up-regulation of 7 genes from *Wnt* signaling and 2 genes associated with epigenetics (*Prmt2* and *Kat6b*), in addition to the down-regulation of 1 gene related to *Wnt* (*Fgf4*) and 15 genes linked to epigenetic modifications

Pathway analyses of the dysregulated genes from the PND5 male mice exposed to e-cig aerosol in utero revealed that they were most significantly associated with *Wnt* signaling (24 genes), regulation of epithelial-mesenchymal transition (EMT) pathway (18 genes), planar cell polarity (PCP) pathway (17 genes), and transforming growth factor-beta (TGF-B) signaling (3 genes) (Fig. 8). The most significantly dysregulated genes included, *Fzd7* (fold-change − 4.4) and *Fzd8* (fold-change − 4.3), both of which are associated with *Wnt* signaling, EMT pathways, and the PCP pathway. Likewise, the genetic dysregulation observed in PND5 females revealed genes that were also associated with these pathways, in addition to 6 genes, including *Gsk3a* (fold-change − 3.3), *Jun* (fold-change − 3.1), and *Ciita* (fold-change − 2.2), involved in pathways associated with the role of nuclear factor of activated T cells (NFAT) in the regulation of the immune response (Fig. 9). Further, at PND11, the gene expression pattern for *Wnt* signaling and epigenetics differed between male and female offspring exposed in utero to e-cig aerosol (Fig. 10). There were only two dysregulated genes in common for both sexes (*Csnk1a1* and *Fzd4*), while we identified 18 and 25 differently expressed genes for males and females, respectively (Fig. 10).

## Discussion

Recent surveys reveal that rates of smoking and vaping during pregnancy are similar and can surpass 7% in the United States [2, 43]. Since the lungs’ functionality associated with gas exchange is centrally dependent on the proper development of alveoli, in the present study, mouse offspring exposed prenatally for 20 consecutive days during gestation were sacrificed at PND5 and PND11 to evaluate how e-cig aerosol exposures affect postnatal lung development, particularly the alveolarization initiation processes. Overall, this study shows that in utero exposures to e-cig aerosols in neonatal mice result in structural and functional impairment of the lungs, as well as dysregulated expression of genes associated with *Wnt* and epigenetics pathways (Fig. 3, 4, 5, 6, 7, 8, 9, 10). Thus, this study provides experimental evidence for future regulations on e-cig products’ use during pregnancy, since in utero e-cig aerosol exposures affect the respiratory system of developing fetuses, with persisting effects at the molecular, structural and functional levels lasting through the alveologenesis period.

Reduced birth weight is a well-known parameter associated with many morbidities—including lung diseases—both immediately and later in life [44–46]. We found that exposures to e-cig aerosol in utero led to a significant decrease in body weights at birth, sustained through PND5 (Fig. 1). These findings support our previously published data [11] and suggest delayed or compromised prenatal physical growth, seen similarly in neonates exposed in utero to a variety of toxins (maternal cigarette smoking, fetal alcohol syndrome, DDT exposure, lead exposure) [47–51]. We also found that the gene expression of inflammation and oxidative stress markers were altered by the in utero e-cig aerosol exposure at PND5 in both male and female offspring (Fig. 4). In early life, inflammation and oxidative stress are processes that have been shown to result in inhibition of alveologenesis, alveolar simplification, and decreased lung function [52, 53]. *Gata3* is a transcription factor associated with type 2 innate lymphoid cells, which produce cytokines including IL-4 and IL-13 [54]. In our study, while *Gata3* expression was up-regulated in e-cig-exposed female offspring (1.6-fold), *Il-4* was down-regulated in both e-cig-exposed male (− 1.7-fold) and female (− 1.8-fold) offspring; *Il-13* was down-regulated (− 1.8-fold) only in males (Fig. 4). It was previously reported that IL-4 and IL-13 are important inducers of goblet cell differentiation during lung organogenesis [21, 55]. Thus, our data indicate that in utero exposures to cinnamon-flavored e-cig aerosol suppress the expression of *Il-4* and *Il-13*, two markers playing key roles in lung maturation, and suggest that the gene expression of *Gata3*, their upstream transcription factor, is increased in efforts to improve type 2 innate lymphoid cells cytokine production. IL-6 is a key cytokine in acute inflammatory reaction; however, it also plays significant roles in cellular proliferation [56, 57]. Studies have shown that IL-6 signaling is crucial in lung maturation processes, as IL-6 is involved in the regulation of pulmonary surfactant proteins, particularly SP-A, which is important for proper lung function [53, 57–59]. Work with rat fetal lung explants demonstrated that inhibiting IL-6 led to considerably decreased branching and decreased cell proliferation [56]. In our study, *Il-6* gene expression was down regulated by − 1.8-fold (Fig. 4) in e-cig-exposed female offspring, suggesting impaired post-natal pulmonary development. In e-cig-exposed male offspring, *Il-10* was down-regulated by 1.5-fold (Fig. 4). IL-10 is an anti-inflammatory cytokine, and its down-regulation may be associated with inefficient regulation of inflammatory processes, thereby increasing the lungs’ vulnerability to incur damage following infection or injury [60]. Lastly, oxidative stress can disturb lung alveolarization as seen in hyperoxia bronchopulmonary dysplasia models [61]. Heme-oxygenase-1 (HMOX1) is an antioxidant defense enzyme for which increased levels were associated with preventing alterations in alveologenesis in bronchopulmonary dysplasia mouse models [61]. Also, increased alveolar size and increased respiratory resistance were observed in *Hmox1* knock out mice [52]. These studies highlight that HMOX1 plays a significant role in lung development, particularly during the alveologenesis period, as alveolar simplification is observed when *Hmox1* is deficient [52, 61, 62]. In our study, *Hmox1* was down-regulated by 1.7-fold (Fig. 4) in e-cig exposed male offspring. This suggests altered alveologenesis. Overall, our data show that in utero exposures to cinnamon-flavored e-cig aerosol can suppress, in a sex-specific manner, the gene expression of inflammation and oxidative stress markers, which play key roles in lung organogenesis, alveologenesis, and maturation processes, and thereby predispose the neonatal lung to compromised function and gas-exchange. In addition, immunosuppression of lung responses following exposures to cinnamon-flavored e-cig aerosols has previously been reported [63].

Another well-known effect of in utero exposures to environmental pollutants is altered lung function. Lung physiological changes have been observed in several epidemiological studies of infants and children exposed to maternal or parental smoking prenatally or in early life [30, 64–66]. Additionally, a study investigating the effect of nicotine on neonatal lungs of non-human primates revealed lung function alterations, including increased pulmonary resistance, decreased lung volume, and decreased peak tidal expiratory flow (FEV_0.2_), similar to the changes observed in children of smokers [67]. This implies that developing lungs exposed to high nicotine concentrations alone (1.5 mg/kg/day, comparable to the nicotine intake level of heavy smokers) can impair pulmonary mechanics postnatally [67]. Moreover, compelling recent published data on humans and various animal models, including mice, rats, sheep, and monkeys, highlight functional lung impairment in offspring exposed in utero to cigarette smoke as mainly driven by nicotine [68]. In the present study, we found that exposure to e-cig aerosol containing 36 mg/mL of nicotine increased the Newtonian resistance, representing the resistance of the conducting airway-of the offspring lungs at PND11 (Fig. 5) [45, 46]. Increased Newtonian resistance is technically an airway effect rather than a peripheral lung effect, and therefore this is a functional measure that would not be impacted directly by the alveoli. The airway resistance values observed in this study (Fig. 5) are in the same range as those previously reported in 14-day old C57BL/6 and Thy-1 null mice [69]. It was previously demonstrated that lung structural changes, including augmented numbers of narrow and smaller airways, are induced by prenatal cigarette smoke exposures in rats [70] and nicotine exposures in mice and lambs [71–73]. Further, a significant increase in respiratory resistance was also measured in 21-day old rats exposed in utero to nicotine [74]. Thus, our data are in line with previous reports and suggest that in utero exposures to nicotine-rich e-cig aerosol may result in lasting lung function limitations, similarly to the effects induced by in utero cigarette smoke or nicotine exposures.

While the exact mechanisms whereby in utero e-cig exposures impair lung function in mid stages of lung alveologenesis (PND11) remain to be elucidated, in addition to anatomical alterations of the lungs’ structure (smaller airways), altered lung function in mice exposed prenatally to cigarette smoke can be caused by increased airway remodeling [71, 72, 75–78]. Extracellular matrix (ECM) remodeling plays a key crucial role in lung alveolar formation, as it facilitates the progression from the saccular to the alveologenesis stages [79]. In mice, PND5 is the onset of lung alveologenesis. It is the period where the lung surface area significantly increases with the development of alveoli via the formation of secondary septae from lung saccules [24]. Secondary septation is a process that originates with the deposition of elastin and collagen by alveolar myofibroblasts [23, 79, 80]. The peak of secondary septation occurs at PND7 and coincides with the peak expression of interstitial collagen [24, 27]. In mice, gene expression of fibrillar collagens (*Col1a1* and *Col3a1*) and basement membrane collagen (*Col4a1* and *Col4a2*) are the highest at PND7 [42]. Further, the quantity of collagen in the lung parenchyma increases as the lung progresses through its various phases of development. The predominant form of collagen found during lung development is fibrillar collagen, in the form of collagen I and III [81, 82]. Here, we found that offspring exposed in utero to e-cig aerosol had a significantly lower percentage of lung fibrillar collagen, measured via SHG microscopy, compared to their in utero air-exposed counterparts at PND5 (Fig. 3). Since collagen provides an interstitial network of fibers necessary for alveolar formation during the secondary septation period, our data suggest that altered lung function measured in the in utero e-cig exposed offspring (Fig. 5) may be associated with impaired ECM support (Fig. 3). This is because ECM provides the typical physical sustenance, and therefore tissue movement, of the airways in formation [83]. Alveologenesis is a dynamically evolving period of lung development, and at this crucial phase, specifically during alveolar septation, lung structural alterations may lead to predictable functional impairments [42, 70–72]. In a recent study, 6-week-old CD-1 offspring mice exposed in utero to e-cig aerosols, with and without nicotine (16%), exhibited (a) dysregulated lung ECM remodeling, as evidenced by increased protein expression of plasminogen activator inhibitor-1 (PAI-1), an essential protein involved in ECM deposition; (b) decreased expression of matrix metalloproteinase 9 (MMP9), a downstream target of PAI-1, as well as (c) altered protein levels of MMP2, COL1A1, and fibronectin (FN1) [84]. These results, along with our data (Fig. 3), support the notion that in utero e-cig aerosol exposures can affect lung ECM remodeling, which, depending on the phase of lung development, can lead to altered lung structure and function in exposed mice offspring. For our study, it is unknown whether the structural and functional effects observed (Fig. 3, 5) are reversible or permanent and whether they could impact the lifelong trajectory of pulmonary health. Several studies, however, on the developmental origin of health and disease indicate that early-life epigenetic effects may play critical roles in adult-onset disorders, including lung diseases [85–90].

Here, we investigated the dysregulation of genes associated with *Wnt* signaling and epigenetic chromatin modifications. The lung gene expression results, mostly assessed for *Wnt* signaling, point to dysregulated EMT, PCP, and TGF-B signaling pathways in both PND5 male and female mice offspring (Fig. 7, 9). The EMT pathway associated with 18 dysregulated genes in males and > 20 dysregulated genes in females at PND5 is vital during lung branching morphogenesis. It allows epithelial cells to adopt a mesenchymal cell phenotype. The EMT pathway requires reciprocated communication between the epithelium and mesenchyme. It is mediated by several pathways—including both the TGF-B and *Wnt* signaling pathways, [91]. At PND5, down-regulated genes in male mice offspring exposed in utero to e-cig aerosol compared to air controls included, *Mapk8* (− 2.2-fold), *Fzd1* (− 2.2-fold), *Fzd7* (− 4.4-fold), *Dvl2* (− 1.7-fold), *Wif1* (− 2.7-fold), and *Wnt11* (− 1.9-fold) (Fig. 6). At this same time-point, in the female mice offspring exposed in utero to e-cig aerosol, down-regulated genes included, *Mapk8* (-twofold), *Fzd1* (− 2.1-fold), *Dvl2* (− 2.5-fold), *Wnt3* (− 1.6-fold), *Wnt5a* (− 1.9-fold), *Wnt5b* (− 5.7-fold), *Wnt7* (− 13.1-fold) and *Wnt8a* (− 3.1-fold) (Fig. 8). In addition, the female offspring exhibited dysregulation of genes associated with the role of NFAT in the regulation of the immune response at PND5. These genes include GSK3A and GSK3B, which are glycogen synthase kinases—some of the most active kinases in cells regulating NFAT at the DNA binding level [92–94]. Similarly, in utero cigarette smoke exposure has been found to cause altered expression of genes associated with NFAT regulation of immune responses in an allergy-induced asthma mouse model [95]. The NFAT pathway, which we found to be highly associated with genes dysregulated in female offspring (Fig. 9), is vital for healthy lung development and neonatal lung function. It contributes to the lungs’ surfactant regulation and morphologic maturation [96, 97]. Abnormal expression of genes associated with the NFAT pathway could be indicative of disrupted pulmonary surfactant homeostasis, which, in turn, could impede lung function in offspring [97]. Thus, down-regulation of these genes (Fig. 6, 8), as well as these aforementioned pathways (Fig. 7, 9), implies interference with lung alveologenesis and maturation processes, whether they are delayed or impaired [9, 32]. In addition, it may be associated with the altered lung structure and impaired lung function we observed in the exposed offspring (Fig. 3, 5). We reported previously that in utero exposure to e-cig aerosol causes the down-regulation of genes associated with *Wnt* signaling at birth [11]. The results of the present study confirm that this alteration is sustained into early life (Fig. 6, 7).

Our data also show that altered gene expression associated with *Wnt* and epigenetics pathways between PND5 and PND11 mainly resolved in neonatal male and female mice exposed in utero to e-cig aerosols (Fig. 6, 8). This suggests that dysregulated expression of genes does not remain static during the first week of alveologenesis in mice. In females, only 10 genes from the *Wnt* pathway were dysregulated at PND11 compared to 61 genes at PND5 (Fig. 8, 10). Common sustained upregulated genes at these two time-points include *Daam1*, *Mmp7*, and *Wnt9a* (Fig. 8). Pathway analysis of these genes revealed associations with collagen formation, collagen degradation, and ECM degradation. The ECM walks a fine line of being both firm and elastic, so alterations to the typical reciprocity between the two may lead to pathological remodeling of the lung tissue [83, 98]. These molecular changes further support our results related to decreased lung fibrillar collagen content at PND5 (Fig. 3) and altered lung function at PND11 (Fig. 5), which were observed in the in utero e-cig aerosol exposed offspring.

Regarding the genes associated with epigenetic chromatin modifications, 60 genes with fold changes ranging from − 11.48 to 3.58 were dysregulated at PND5. In comparison, 17 genes, with fold-changes ranging from − 2.85 to 1.70, were dysregulated at PND11 in the e-cig exposed female mice offspring (Fig. 8, 10). In males, only one gene, *Hdac9*, was significantly upregulated at PND5, while four epigenetic-related genes were dysregulated at PND11 (Fig. 6, 10). Conserved down-regulated genes between the two time-points in female offspring include *Esco1*, *Aurkc,* and *Hdac3* (Fig. 8, 10). Dysregulation of those three epigenetic chromatin modification genes has been associated with asthma and emphysema pathogenesis in humans and in experimental studies [99–102]. Further, it is well-established that DNA methylation patterns can heighten an individual’s risk for chronic disease, e.g., asthma and COPD, and previously have been linked to in utero and/or postnatal exposure to tobacco smoke in various human studies [12–15, 103]. In the present study, pathway analyses revealed functional clusters enriched for hydrolase activity (enrichment score of 4.59 for the gene cluster including *Hdac3*, *Hdac5*, *Hdac8*, *Hdac9*, *Mmp7,* and *Mysm1*) in the e-cig-exposed female mice offspring at PND11. Increased hydrolase activity in the lungs has been associated with the pathogenesis of chronic lung diseases [104]. Overall, at the molecular level, female mouse offspring were more affected than the male offspring by the in utero e-cig exposures, at the two-time points evaluated—PND5 and PND11 (Fig. 6, 8). At PND11, however, 17 epigenetic-related lung genes were dysregulated in the female mice offspring compared to only four lung genes in males (Fig. 10). This suggests that female mouse offspring exposed in utero to e-cig aerosols may be less protected at the epigenetic level against an increased risk of developing lung diseases later in life. Indeed, since there are more epigenetic chromatin modification genes that are dysregulated in females than in males (Fig. 10), this implies that the methylation status of specific genes are altered, potentially resulting in the development of adverse health effects at older ages in female mice. This is in line with what is observed in children, with a 3.4% increase in methylation found on the promoter region of AXL (receptor tyrosine kinase), a protein-coding gene analyzed in buccal cells of 11-year-old girls exposed in utero to maternal smoking compared to boys [105]. Furthermore, DNA methylation of AXL at birth has been associated with an increased risk of developing asthma-related responses in childhood, with girls being more affected than boys [106]. Our data also is supported by the higher prevalence of asthma in girls/women during both adolescence and adulthood (e.g., later in life), in addition to in utero exposures to maternal smoking and SHS being demonstrated risk factors for asthma development in exposed offspring [107]. In contrast, since very few (< 5) epigenetic-related genes are dysregulated in male mouse offspring (Fig. 10), males may be more prone to develop immediate/early life pulmonary effects, as supported by a higher prevalence of childhood asthma and respiratory infections observed in infant boys [107–110].

Slight temporal differences in lung development of male and female fetuses have been reported [111]. For instance, the synthesis of the pulmonary surfactant as well as processes related to lung maturation was found to occur earlier in female fetuses than in male fetuses [111]. This could possibly explain the difference in NFAT regulated immune response among the sexes, as mentioned above. Additionally, a greater number of genes associated with *Wnt* signaling were found to be down-regulated in female than in male mice, which could also be related to altered surfactant regulation, being that *Wnt* signaling inhibition hinders the differentiation of alveolar epithelial cells to the AT2 cell phenotype [25, 28]. Overall, we observed sex-specific differences in lung transcriptomic data in the in utero e-cig exposed offspring at PND5 and 11 (Fig. 6, 7, 10). This observation is supported by recently published literature that determined that in utero e-cig exposures affected offspring in a sex-linked manner [84].

Taken together, our physiological and transcriptomic results, with datasets identifying sex-specific molecular signatures imprinted by in utero e-cig exposures on the lung, combined with our previously published data [11] lay the groundwork for studies investigating the mechanisms by which in utero e-cig aerosol exposures impair lung development, during both organogenesis (in utero) and alveologenesis (postnatal) periods in mice. These mechanisms could involve EMT, PCP, and TGF-B signaling pathways, all playing critical roles in lung branching morphogenesis (Fig. 6, 7, 8, 9, 10). Thus, this study exhibits several strengths, but also has a few limitations, which could constrain the generalizability and inference of the data presented. First, the results we obtained were from mice exposed in utero to cinnamon-flavored e-cig aerosol containing 36 mg/mL of nicotine that was produced by a third-generation e-cig device. Since e-cig aerosol toxicity is specific and highly dependent on the composition of the e-liquid and on the e-cig device used, it is unclear how in utero exposures to differently flavored e-cig aerosols containing lower nicotine concentrations would compare. Second, while all offspring were exposed in utero during the various stage of lung development [(lung embryonic phase (E8-9.5), pseudoglandular stage (E9.5–16.5), and canalicular stage (E16.5–17.5)] before birth, 5 out of 6 air group litters and 4 out of 7 e-cig group litters were also exposed during early parts of the saccular stage, starting at E.17.5. The other litters from both groups were exposed up to gestational days 16 or 17. This could have increased the inter-subject variability in the e-cig exposed group, making it more difficult to observe statistically significant differences between the air- and e-cig-exposed groups. Finally, it is well-known that there are sex-specific differences in lung function from birth to adulthood. Although it is common practice to assess lung function of mouse pups at a young age (PND5 and 11) on both sexes to determine a group or treatment effect, these lung function results could be confounded by the sex of the animal. Therefore, the lung function results should be interpreted with caution, as we established a group effect rather than groups per sex effects.

In summary, our results indicate that in utero exposure to e-cig aerosols can significantly decrease birth weight through PND5 (Fig. 1). This is accompanied by reduced lung fibrillar collagen content (Fig. 3) in addition to declines in offspring lung function at PND11 (Fig. 5). Thus, our data suggest that in utero exposures to e-cig aerosol inhibit normal alveologenesis processes and affect postnatal lung maturation processes (Fig. 3, 4, 5, 6, 7, 8, 9, 10). Additionally, during the onset of alveologenesis, we found that in utero exposures to e-cig aerosols induced sex-specific molecular changes associated with *Wnt* signaling and epigenetic modifications at PND5, the latter of which was highly conserved through PND11 in females. This indicates that in utero exposures to e-cig aerosols result in lasting molecular changes through at least PND11 of neonatal life (Fig. 6, 8), which may negatively impact lung function later in life (both in childhood and adulthood).

These experimental data strongly suggest that maternal e-cig use negatively affects the fetus and therefore impedes the maturation of neonates, specifically by delaying and/or impairing the postnatal development of the respiratory system. Molecular signatures and altered lung function induced by in utero e-cig aerosol exposures may serve as potential markers of impaired lung development in exposed offspring. Investigating the epigenetic patterns in the lungs modified by in utero e-cig exposures can help further our understanding of the potential development of associated lung disease and related preventive measures. In addition, the present study indicates that e-cigs should not be viewed as a “safe alternative” to tobacco smoking, especially for high-risk populations such as expectant mothers and their fetuses. Further research is required to reveal long-term health effects and possible heightened disease susceptibility caused by in utero e-cig exposures.
